# Pro-Inflammatory Profile of Preeclamptic Placental Mesenchymal Stromal Cells: New Insights into the Etiopathogenesis of Preeclampsia

**DOI:** 10.1371/journal.pone.0059403

**Published:** 2013-03-19

**Authors:** Alessandro Rolfo, Domenica Giuffrida, Anna Maria Nuzzo, Daniele Pierobon, Simona Cardaropoli, Ettore Piccoli, Mirella Giovarelli, Tullia Todros

**Affiliations:** 1 Department of Surgical Sciences, University of Turin, Turin, Italy; 2 Department of Obstetrics and Neonatology, O.I.R.M. S. Anna Hospital, Turin, Italy; 3 Department of Molecular Biotechnology and Health Sciences, University of Turin, Turin, Italy; 4 Center for Experimental Research and Medical Studies (CERMS), San Giovanni Battista Hospital, Turin, Italy; Georgia Regents University, United States of America

## Abstract

The objective of the present study was to evaluate whether placental mesenchymal stromal cells (PDMSCs) derived from normal and preeclamptic (PE) chorionic villous tissue presented differences in their cytokines expression profiles. Moreover, we investigated the effects of conditioned media from normal and PE-PDMSCs on the expression of pro-inflammatory Macrophage migration Inhibitory Factor (MIF), Vascular Endothelial Growth Factor (VEGF), soluble FMS-like tyrosine kinase-1 (sFlt-1) and free β-human Chorionic Gonadotropin (βhCG) by normal term villous explants. This information will help to understand whether anomalies in PE-PDMSCs could cause or contribute to the anomalies typical of preeclampsia.

**Methods:**

Chorionic villous PDMSCs were isolated from severe preeclamptic (n = 12) and physiological control term (n = 12) placentae. Control and PE-PDMSCs’s cytokines expression profiles were determined by Cytokine Array. Control and PE-PDMSCs were plated for 72 h and conditioned media (CM) was collected. Physiological villous explants (n = 48) were treated with control or PE-PDMSCs CM for 72 h and processed for mRNA and protein isolation. MIF, VEGF and sFlt-1 mRNA and protein expression were analyzed by Real Time PCR and Western Blot respectively. Free βhCG was assessed by immunofluorescent.

**Results:**

Cytokine array showed increased release of pro-inflammatory cytokines by PE relative to control PDMSCs. Physiological explants treated with PE-PDMSCs CM showed significantly increased MIF and sFlt-1 expression relative to untreated and control PDMSCs CM explants. Interestingly, both control and PE-PDMSCs media induced VEGF mRNA increase while only normal PDMSCs media promoted VEGF protein accumulation. PE-PDMSCs CM explants released significantly increased amounts of free βhCG relative to normal PDMSCs CM ones.

**Conclusions:**

Herein, we reported elevated production of pro-inflammatory cytokines by PE-PDMSCs. Importantly, PE PDMSCs induced a PE-like phenotype in physiological villous explants. Our data clearly depict chorionic mesenchymal stromal cells as central players in placental physiopathology, thus opening to new intriguing perspectives for the treatment of human placental-related disorders as preeclampsia.

## Introduction

Preeclampsia (PE) is a severe placenta-related syndrome exclusive of human pregnancy that represents the main cause of feto-maternal mortality and morbidity worldwide [1], [2]. PE affects 5–10% of all pregnancies [1], [2] and, despite intensive investigation over the past decade, its etiopathogenesis still remains elusive. Clinical features of preeclampsia are severe maternal hypertension accompanied by maternal and placental exacerbated inflammatory response and generalized endothelial damage [3], [4]. Even though PE resolves with placenta removal, it may cause long term complications as hypertension, cardiovascular diseases, metabolic and neurological disorders for both the mother and the newborn [5]. PE clinical symptoms become evident in the third trimester of pregnancy, but it is widely believed that they originate from anomalies in placenta development earlier on during first trimester. The PE placenta is characterized by immature trophoblast phenotype with shallow invasion of maternal spiral arteries and impaired villous vasculogenesis [6]. These aberrations lead to reduced utero-placental perfusion and placental ischemia, with consequent increased systemic release of pro-inflammatory cytokines and anti-angiogenic factors that promote endothelial cells activation and damage [6], [7], [8], [9]. Maternal immune maladaptation towards the feto-placental district has been implicated as a possible cause for the defective trophoblast development and related maternal-placental pathological anomalies.

The trophoblast is generally considered the main placental site of the morphological and molecular alterations responsible for the onset of preeclampsia. Nevertheless, the human placenta is a heterogeneous organ composed of several cellular populations each contributing to the organ physiology. Indeed, beside the trophoblast, placental villi are composed of endothelial cells and progenitors, myofibroblasts and mesenchymal stromal cells forming a complex network that concertedly interacts to maintain placental functionality.

Recently, the human placenta has been identified as source of a particular type of mesenchymal stromal cells (MSCs) that retain stem cell-like properties. Placental MSCs (PDMSCs) have been successfully isolated from the placental basal plate, the amnion and the decidua. They are characterized by different degrees of plasticity and possess unique immunologic and immune-regulatory properties [10], [11]. MSCs do not express HLA-DR [12] and have the ability to suppress the “mixed lymphocyte reaction”, thus being non-immunogenic and exerting an immunosuppressive effect on T-cells [12]. Recently, amniotic MSCs have been shown to promote angiogenic growth and to possess anti-inflammatory and anti-fibrotic activities mediated by the release of specific trophic factors [13].

MSCs plasticity is of great interest for regenerative medicine and several groups are exploring the possibilities offered by the placenta as a new therapeutic tool. On the other hand, the investigation of placental MSCs immune-modulatory, pro-angiogenic and anti-inflammatory properties could open new perspectives into the understanding of placenta-related disorders due to maternal immune maladaptation and aberrations of trophoblast development as preeclampsia. The secretion of cytokines and chemoattractant by the first trimester placenta with consequent recruitment of lymphocytes, natural killer cells and macrophages is necessary to maintain proper pregnancy physiology [14], [15], [16], [17]. Nevertheless, the aberrant production of inflammatory molecules by placental cells could result in excessive recruitment of macrophages and immune cells leading to the abnormal placental development, inflammation and endothelial dysfunction typical of PE. Indeed, pregnancies complicated by preeclampsia and Fetal Growth Restriction are characterized by increased presence of aberrantly-activated macrophages into the placental bed, data that were correlated with reduced trophoblast invasion [18].

Our hypothesis is that anomalies in PDMSCs anti-inflammatory and pro-angiogenic properties might cause or contribute to the defective placental development and/or immune maladaptation typical of preeclampsia”. In the present study, we characterized cytokine expression profiles of PDMSCs isolated from the basal plate of normal and severe PE placentae. Moreover, we investigated the effect of conditioned media from normal and PE PDMSCs on the expression of Macrophage migration Inhibitory Factor (MIF), Vascular Endothelial Growth Factor (VEGF), soluble FMS-like tyrosine kinase-1 (sFlt-1) and β-human Chorionic Gonadotropin (βhCG) in normal placental villous explants. MIF is a pro-inflammatory cytokine key player in PE inflammatory response [19], while VEGF is the main pro-angiogenic factor sequestered and down-regulated by sFlt-1, soluble variant of VEGF receptor 1 which aberrant expression is an hallmark typical of PE [20], [21], [22], [23], [24], [25]. Thus, we will determine whether PE PDMSCs could negatively influence trophoblast inflammatory response and angiogenesis.

## Materials and Methods

### Ethics Statement

This study was conducted according to the principles expressed in the Declaration of Helsinki. The study was approved by the Institutional Review Board of O.I.R.M. S.Anna Hospital and “Ordine Mauriziano di Torino” (n.209; protocol 39226/C.27.1 04/08/09) (Turin, Italy). All patients provided written informed consent for the collection of samples and subsequent analysis.

### Tissue Collection

The study groups included singleton pregnancies complicated by severe preeclampsia (n = 12) and physiological control term pregnancies (n = 12). The diagnosis of PE was made according to the following criteria: presence of pregnancy-induced hypertension (systolic ≥140 mmHg, diastolic ≥90 mmHg) and proteinuria (≥300 mg/24 h) after the 20^th^ weeks of gestation in previously normotensive women [2]. We excluded pregnancies with congenital malformations, chromosomal anomalies (of number and/or structure) or evident intra uterine infections. Physiological control placentae were obtained from normal pregnancies that did not show any signs of preeclampsia or other placental disease. Patients with diabetes, infections, kidney disease, congenital malformations and chromosomal anomalies (number and/or structure) were excluded. Placental villous samples were collected randomly from the central placental area and snap frozen immediately after delivery. Calcified, necrotic and visually ischemic areas were excluded from collection.

### Placenta-derived Mesenchymal Stromal Cells (PDMSCs) Isolation

PDMSCs were isolated by using a protocol modified from Brooke and colleagues [26]. Immediately after delivery, the amniotic membranes were mechanically removed and the decidua was peeled off from the placental basal plate and discharged, in order to avoid maternal cells contamination. From each control and PE placenta, 30 grams of tissue were excised from the central part of placental cotyledons, thus avoiding decidual contamination from the placental septa, and washed several times with Hank’s Buffered Salt Solution (HBSS, Gibco, Life Technoligies, Italy) to remove excess of blood. Next, the placental tissue was mechanically minced and incubated with 100 U/ml collagenase type I (Gibco, Life Technologies, Italy) plus 5 µg/ml DNAse I (Invitrogen by Life Technologies, Italy) for 3 hours. After enzymatic digestions, the cells were separated by gradient using 1.073 Ficoll Paque Premium (GE Healthcare Europe, Italy) and the mononuclear cells ring was collected, washed and cells were resuspended in Dulbecco’s modified Minimum Essential Medium (DMEM, Gibco, Life Technoligies, Italy) supplemented with 10% Fetal Bovine Serum (FBS Australian origin, Italy) and seeded in T150 flask. Importantly, the culture media was not supplemented with basic fibroblast growth factor (bFGF) in order to maintain the original biochemical and molecular features of the placenta-derived mesenchymal cells. The cultures were maintained at 37°C and 5% CO_2_.

### PDMSCs Characterization

After passages three to five, the cells were evaluated for specific mesenchymal stromal cells antigens by flow cytometry. The following markers were investigated: HLA-DR, CD105, CD166, CD90, CD34, CD73, CD133, CD20, CD326, CD31, CD45, CD14. All the antibodies were purchased from Myltenyi Biotec (Myltenyi Biotec, Bologna, Italy). Moreover, Oct4 and Nanog mRNA expression levels were assessed in both control and PE PDMSCs by semi-quantitative RT PCR in order to verify cellular stemness. Primers sequences were the following: Oct4 FW(5′-CGT GAA GCTG GAG AAG GAG AAG CTG-3′) RV(5′-CAA GGG CCG CAG CTT ACA CAT GTT C-3′); Nanog FW(5′-AAT ACC TCA GCC TCC AGC AGA TG-3′) RV(5′-CTG CGT CAC ACC ATT GCT ATT CT-3′).

### 3-(4,5-dimethylthiazol-2-yl)-2,5-diphenyl-2H-tetrazolium Bromide (MTT) Assay

Normal and preeclamptic PDMSCs proliferation ability was assessed by MTT assay (Sigma, Italy, Cat.no. M5655). At passage five, physiological (3 different cell lines) and PE (3 different cell lines) PDMSCs were seeded in 96-well plates at a density of 6000 cells per well. Each cell lines was plated in six different wells. At time 0, 48 h and 120 h of culture, cells were incubated with 5 mg/ml of MTT for 4 h. Next, medium was removed and formazan salts were dissolved with 100 µl of dimethylsulfoxide (DMSO, Sigma, Italy Cat. no 000070). 570 nm absorbance at each time point was determined using a microplate spectrophotomether in order to establish PDMSCs cell proliferation curve.

### Determination of Cellular Senescence

Senescence-associated β-galactosidase (SA-b-gal) nuclear staining was performed using a SA-b-gal staining kit (Sigma, Cat.no.CS0030) following manufacturer instructions. At passage five, normal (two different cell lines) and PE (2 different cell lines) PDMSCs were plated in 6 well plates at a density of 2×10^5^ cells/well in DMEM LG supplemented with 10% FBS. Each cell line was seeded in six different wells. After 48 h of culture, the cells were fixed in 1X Fixation Buffer for 6 minutes and were stained with SA-b-gal–staining solution for 4 h. The senescent SA-b-gal–positive cells exhibited a blue nuclear color. Number of positive cells number was determined by phase-contrast microscopy.

### Conditioned Media (CM) Preparation

At passage five, PDMSCs were plated in 6 well plates at a density of 2.0×10^5^ cells/ml (final volume: 2 ml, 4×10^5^ cells total). After 72 h of culture, conditioned media was collected, centrifuged, filtered to remove cellular debries and immediately stored at −80°C, while cells were processed for mRNA and protein isolation.

### Cytokines Array

To investigate differences in cytokines production between normal and preeclamptic PDMSCs, the RayBio Human Cytokine Antibody Array (#AAH-CYT-5, RayBiotech Inc, GA, USA) was used. This specific array was chosen because it allows contemporary detection and quantification of eighty different human cytokines, chemokines and inflammation-related growth factors. Arrays were performed on unconditioned and conditioned culture media from normal and PE PDMSCs (prepared as described above) following manufacturer instructions. Cytokines levels were quantified by densitometric analysis using ImageQuant software and normalized by setting the positive controls as 100 and negative controls as zero percent. The arrays were performed in duplicate on CMs derived from four normal term PDMSCs lines, four preeclamptic PDMSCs lines and on unconditioned media (UCM) as baseline for molecules already present in the culture media. UCM data were used to normalize Normal and PE PDMSCs CM results. Cell lines used were all derived from different control and PE patients.

### Human Chorionic Villous Explants Cultures and PDMSCs Conditioned Media Treatment

Placental biopsies from physiological term placentae were processed within 2 hours from delivery. Fetal membranes and decidua were mechanically removed and placental tissues were washed in phosphate-buffered saline (PBS) solution to remove excess of blood. Small portions of placental chorionic villi (35 mg, n = 48) were excised and placed in a 24-well culture dish. Explants were cultured in Ham’s F12 media (Gibco, Invitrogen by Life Technologies, Italy) and incubated overnight at 37°C and 5% CO_2_ to equilibrate. Next, culture media was removed and explants were treated for 72 hours with 500 µl of conditioned media from 4 different control PDMSCs lines (n = 16 explants) and 4 different PE PDMSCs lines (n = 16 explants). Explants treated by unconditioned culture media were used as controls (n = 16 explants). Finally, culture media were collected while control and treated explants were processed for mRNA and protein isolation. Each experimental condition was performed in duplicate.

### RNA Isolation and Real Time PCR

Total RNA was extracted from placental MSCs and chorionic villous explants using TRIZOL reagent according to manufacturer instructions (Invitrogen by Life Technologies, Italy) and treated with DNAse I to remove genomic DNA contamination. Two µg of total RNA was reverse transcribed using random hexamers approach (Fermentas Europe, St. Leon-Rot., Germany). The resulting templates were quantified by Real-time PCR (StepOne™ Real-Time PCR System, Applied Biosystems, Carlsbad, California). TaqMan primers and probes for ribosomal 18S, MIF, VEGF, HCGB, IL-8, IL-6 and TNF-α were purchased from Applied Biosystems as TaqMan® Gene Expression Assays. sFlt-1 primers and probe were designed as previously described by Nevo and colleagues [23] and purchased from Applied Biosystems as Custom Gene Expression Assays. For the relative quantitation, PCR signals were compared among groups after normalization using 18S as internal reference. Relative expression and fold change was calculated according to Livak and Schmittgen [27].

### Western Blot Analysis

Total proteins were isolated from PDMSCs and chorionic villous explants using 1X Radio Immuno-precipitation Assay (RIPA) buffer. Fifty µg of total protein from PDMSCs and villous explants were processed by SDS-page electrophoresis on 4–12% polyacrylamide pre-cast gradient gels (Bio-Rad Laboratories S.r.l., Italy). Next, proteins were transferred on Polyvinylidene fluoride (PVDF) membranes and probed at room temperature with primary antibodies using the SnapID system (Merk-Millipore, Italy) following manufacturer instructions. Primary antibodies were: mouse monoclonal anti-human MIF (1∶1000 dilution, R&D Systems, MN, USA), rabbit polyclonal anti-human VEGF (1∶1000 dilution, R&D Systems, MN, USA), rabbit polyclonal anti-human soluble VEGF Receptor (sFlt-1, 1∶250 dilution, Life Technologies-Invitrgen, Italy), rabbit polyclonal anti-human Ki67 (1∶500 dilution, Santa Cruz Biotechnologie, USA), goat polyclonal anti-human Cytokeratin 7 (1∶500 dilution, Santa Cruz Biotechnologie, USA), mouse monoclonal anti-human CD45 (1∶500 dilution, Santa Cruz Biotechnologie, USA) and mouse monoclonal anti-human CD166 (1∶200 dilution, Santa Cruz Biotechnologie, USA). Biotinylated secondary antibodies were goat anti-mouse for MIF, CD45 and CD166 (1∶1000 dilution, Vector Laboratories, UK) and donkey anti-rabbit for VEGF, sFlt-1 and Ki67 (1∶1000 dilution, Vector Laboratories, UK) and donkey anti-goat for Cytokeratin 7 (1∶100 dilution, Santa Cruz Biotechnologie, USA). Blots were visualized using the QDot reagent kit (Invitrogen by Life Technologies, Italy) following manufacturer instructions.

### Free βhCG Assay

Free β hCG released by physiological villous explants treated with normal or PE PDMSCs conditioned media was measured using the B·R·A·H·M·S Free βhCG KRYPTOR immunofluorescent assay (Thermo Scientific, Germany) following manufacturer instructions.

### TNF-α and VEGF Enzyme-linked Immunosorbent Assays (ELISA)

Concentrations of TNF-α and VEGF proteins in normal and PE-PDMSCs CM and in protein lysates of CM-treated villous explants were evaluated by commercially available ELISA kits (Peprotech), according to the manufacturer’s recommendations. Values are given as the mean concentration ± SEM of fourteen (PDMSCs CM) and twelve (CM-treated villous explants) independent experiments, all repeated in duplicate.

### Statistical Analysis

All data are represented as mean ± SE. For comparison of data between multiple groups we used one-way analysis of variance (ANOVA) with posthoc Dunnett’s test. For comparison between 2 groups we used paired and unpaired Student’s t-test as appropriate. Statistical test were carried out using Graph Pad Prism 5 statistical software and significance was accepted at P<0.05.

## Results

### Study Population

Clinical data of the study population are summarized in Table 1. Seventy-five percent of the babies from severe preeclamptic pregnancies were growth restricted (Table 1), while 44% of PE pregnancies were characterized by abnormal Doppler flow velocity waveforms in the umbilical arteries (AEDF or REDF) and 88% of them had pathological uterine Doppler.

**Table 1:** Clinical Features of the Study Population.

	Controls (n = 12)	Severe Preeclampsia (n = 12)
**Mean Maternal Age (yr)**	32.4±4.72	34.75±5.59
**Mean Gestational Age at delivery** **[wk (range)]**	39.77±0.85 (38–41)	32.96±3.97 (28–41)
**Blood Pressure (mmHg)**	Systolic: 123±11.83	Systolic: 151±17.99
	Diastolic: 77±9.77	Diastolic: 91.41±11.56
**Proteinuria (g/24** **h)**	Absent	1.40±2.16
**Fetal Weight (g)**	A.G.A. (n = 12): 3671.5±345.29	A.G.A.(n = 3): 2710±1411.70
		IUGR (n = 9): 1204.77±473.74
**IUGR**	0%	75%
**A/REDF (% of women)**	0%	44%
**Pathological Uterine Doppler R.I**	0%	88%
**Fetal Sex**	Males = 70%	Males = 41%
	Females = 30%	Females = 59%
**Mode of delivery**	CS 45%	CS 83%

**A.G.A.:** Appropriate for Gestational Age; **IUGR:** Intra Uterine Growth Restriction; **CS:** Caesarean Section.

### Placenta-derived Mesenchymal Stem Cells Showed Proper MSC Antigens Profile

PDMSCs were isolated from both physiological term and preeclamptic placentae and, starting from passages 3 to 5, they were characterized for the expression of typical MSCs markers by flow cytometry. All PDMSCs lines investigated were positive for CD105, CD166, CD90 and CD73, while they were negative for HLAII, CD34, CD133, CD20, CD326, CD31, CD45 and CD14 (Fig. S1A), thus showing proper mesenchymal stem cell phenotype and excluding contamination from trophoblast/epithelial cells and hematopoietic progenitors. Moreover, all PDMSCs properly expressed both Oct4 and Nanog mRNA (Fig. S1B).

### PDMSCs Isolated from PE Placentae Showed Decreased Proliferation and Increased Cellular Senescence Relative to Normal PDMSCs

**Figure 1:**
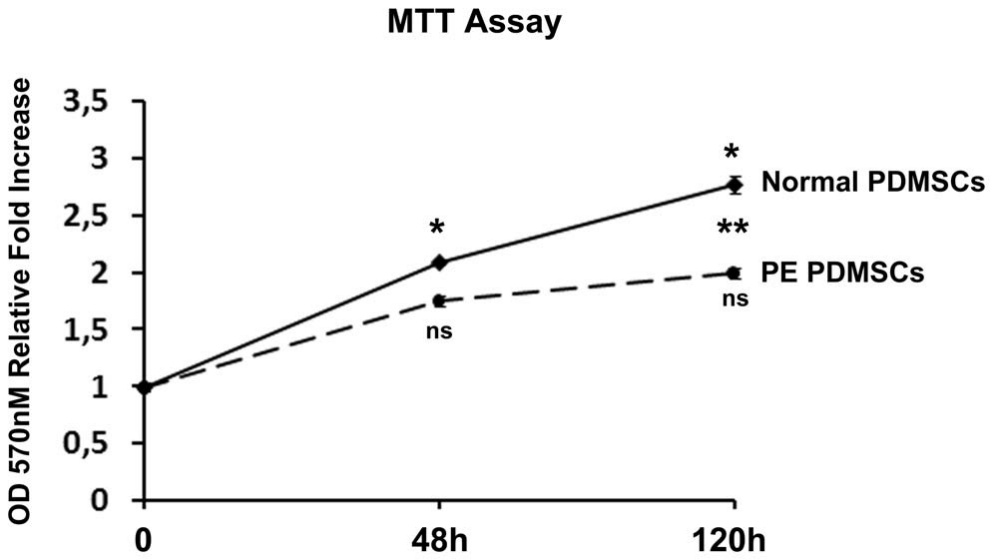
Cell proliferation rate in Normal and PE-PDMSCs. Cell proliferation in Normal and PE-PDMSCs was assessed by MTT assay at time 0, 48 and 120 hours of culture. Results are expressed as means ± SE of six independent samples. Statistical significance has been considered as p<0.05. (*) = statistical significance among Normal PDMSCs time points; (**) = statistical significance between Normal and PE-PDMSCs at 120 hours of culture. (ns) = not statistically significant, referred to comparisons among PE PDMSCs time points.

**Figure 2:**
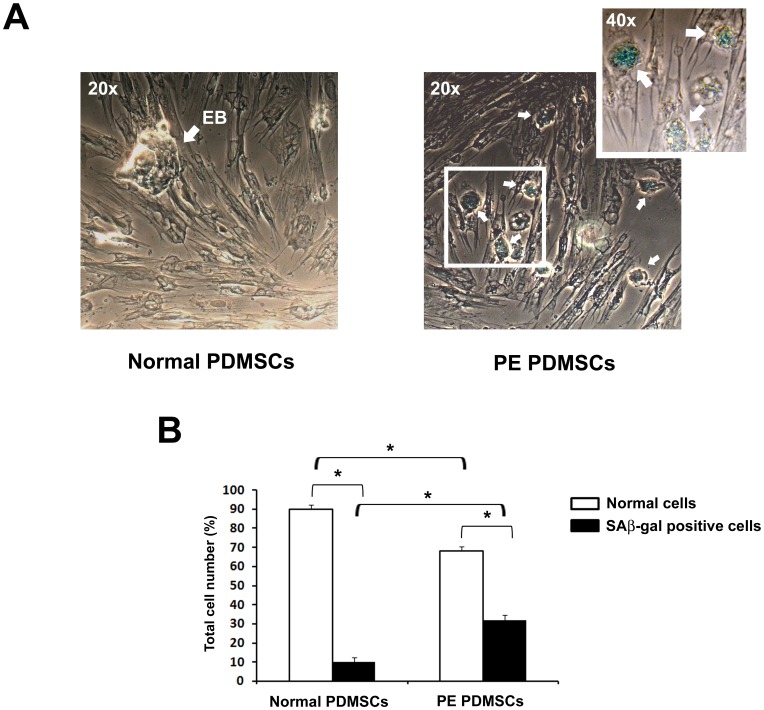
Senescence quantification in PDMSCs isolated from normal and preeclamptic placentae. (A) Normal (left panel) and PE (right panels) PDMSCs representative images after SAβ-gal nuclear staining. EB: Embryoid Body. White Arrows indicate blu nuclei positive for SAβ-gal staining, indicative of cellular senescence. (B) Quantitative analysis of cellular senescence in Normal and PE-PDMSCs. For quantitative analysis, SAβ-gal positive cells were considered to be senescent cells. The percentages of senescent cells after 48 h of incubation were determined in Normal and PE-PDMSCs populations. At least 200 cells were counted in each group. Results are expressed as means ± SE of six independent samples. Statistical significance (*) has been considered as p<0.05.

PDMSCs cell proliferation at 48 h and 120 h was evaluated by MTT assay. We reported a significant cell proliferation increase in Normal PDMSCs at both 48 h (p<0.01, 2 Fold Increase) and 120 h (p<0.01, 1.4 Fold Increase vs 48 h, 2.7 Fold Increase vs time 0), whereas PE PDMSCs showed a slower and not significant proliferation rate at both 48 h and 12 h (Figure 1). Importantly, despite Normal and PE PDMSCs had the same cell number at time 0, cell proliferation at 120 h, as determined by MTT assay, was significantly increased in Normal vs PE PDMSCs (p<0.01, 1.4 Fold Increase, Figure 1). We next performed senescence-associated β-galactosidase (SA-b-gal) staining in normal and preeclamptic PDMSCs. We found significantly increased percentage of positive/senescent cells in PE relative to normal PDMSCs (p<0.01, 3.2 Fold Increase, Figure 2B).

### Cytokines Expression Profiles in Conditioned Media from Normal and Preeclamptic PDMSCs

**Figure 3:**
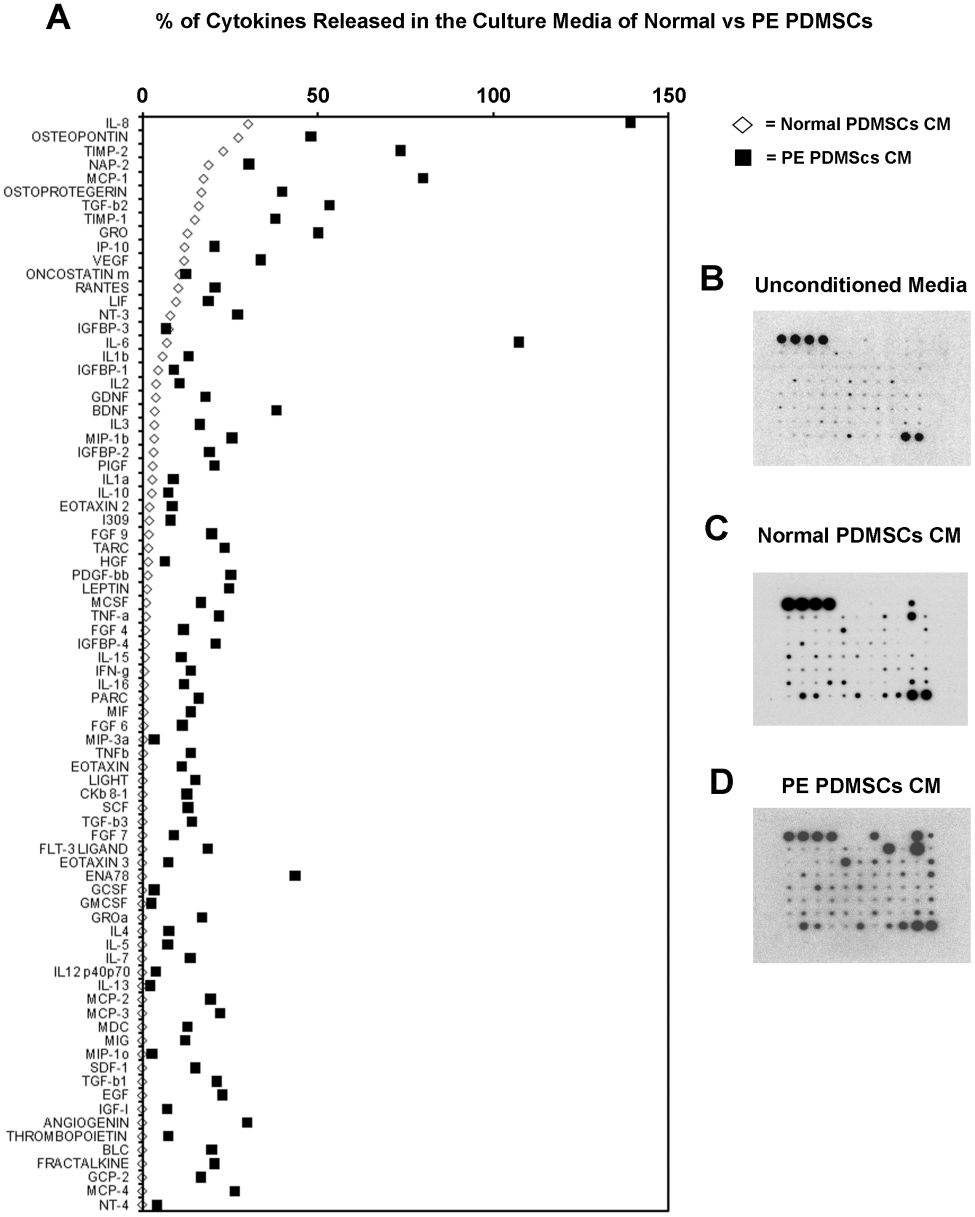
Expression of pro-inflammatory cytokines, chemokines and growth factors in control and PE PDMSCs conditioned media. A) Scatter plot showing increased release in the culture media of pro-inflammatory cytokines and chemokines (80 molecules analyzed) by PE relative to control PDMSCs. Each data point represent the average of four control media from normal PDMSCs (white squares) and four PE PDMSCs media (black squares) used for the analysis. B) Representative cytokine array blots of unconditioned media (upper panel) and media conditioned by control (middle panel) and PE (lower panel) PDMSCs.

Mesenchymal stem cells are a promising tool for regenerative medicine because of their unique immunomodulatory, pro-vasculogenic and anti-inflammatory properties. Since preeclampsia is characterized by an exacerbated feto-maternal inflammatory response accompanied by generalized endothelial damage and aberrant placental vascular development, we investigated whether PDMSCs derived from normal and PE placentae showed differences in cytokines production profiles. These data would help us to understand whether or not PDMSCs could contribute to physiological placenta development and/or to the aberrant placentation and inflammatory response typical of PE. To reach our goal, we performed a cytokine array able to detect 80 different cytokines (Table S1) present in the conditioned media of both control and PE PDMSCs. Cytokines expression levels in Normal and PE PDMSCs conditioned media were compared to positive and negative standards (used as 100% and 0% expression ranges respectively) (Figure 3A). The results reported in Table S1 and in Figure 3 clearly demonstrated that PDMSCs from normal and PE placentae posses different cytokine expression profiles. Interleukine 8 (IL-8), Interleukine 6 (IL_6), Epithelial neutrophil-activating protein 78 (ENA-78), Transforming Growth Factor-β2 (TGF-β2), Monocyte Chemotactic Protein-1 (MCP-1), Tissue Inhibitor of Metalloproteinases-2 (TIMP-2), Brain-derived neurotrophic factor (BDNF), Vascular Endothelial Growth Factor (VEGF), Angiogenin, IGF binding protein 4 (IGFBP-4), Macrophage Inflammatory Protein-1β (MIP-1β), Platelet-derived Growth Factor BB (PDGF-BB), MCP-3, Thymus and Activation Regulated Chemokine (TARC), Tumor Necrosis Factor-α (TNF-α), Leptin, Fibroblast Growth Factor-9 (FGF-9), Osteopontin, and Osteoprotegerin were dramatically more abundant (differences higher than 19%) in PE relative to Normal PDMSCs conditioned media.

In addition, Interferon-inducible protein-10 (IP-10), Leukemia inhibitory factor (LIF), Neurotrophin-3 (NT-3), Neutrophil-activating Protein-2 (NAP-2), Macrophage-migration Inhibitory Factor (MIF), Placental Growth Factor (PlGF) and Pulmonary and Activation-regulated Chemokine (PARC) were markedly increased (range of increase: 8–19%) in preeclamptic PDMSCs conditioned media (Figure 3, Table S1).

Furthermore, we performed Real Time PCR analysis on normal and PE-PDMSCs in order to confirm increased expression of IL-8, IL-6, MIF, TNF-α and VEGF as detected by Cytokine Array analysis (Figure S2). We selected this specific pool of molecules because they represent key players in PE pathogenesis (MIF, TNF-α and VEGF) and the main pro-inflammatory cytokines over-expressed by PE-PDMSCs (IL-8 and IL-6). We found significantly increased expression of MIF (p<0.001, 2.9 Fold Increase), VEGF (p<0.01, 1.9 Fold Increase), TNF-α (p<0.01, 2 Fold Increase), IL-8 (p = 0.02, 2.3 Fold Increase) and IL-6 (p = 0.02, 2.2 Fold Increase), thus confirming Cytokine Array results (Figure S2). Finally, we further corroborated our findings on PE-PDMSCs increased TNF-α and VEGF protein release by using specific ELISA assays. We found that PE-PDMSCs CM contains significantly higher concentrations of both TNF-α (p<0.01, 34 Fold Increase) and VEGF (p = 0.02, 1.55 Fold Increase) molecules relative to normal PDMSCs CM (Figure S3A).

### Conditioned Media from Preeclamptic PDMSCs Induced MIF, VEGF, sFlt-1 and Ki67 Over-expression in Physiological Human Villous Explants

**Figure 4:**
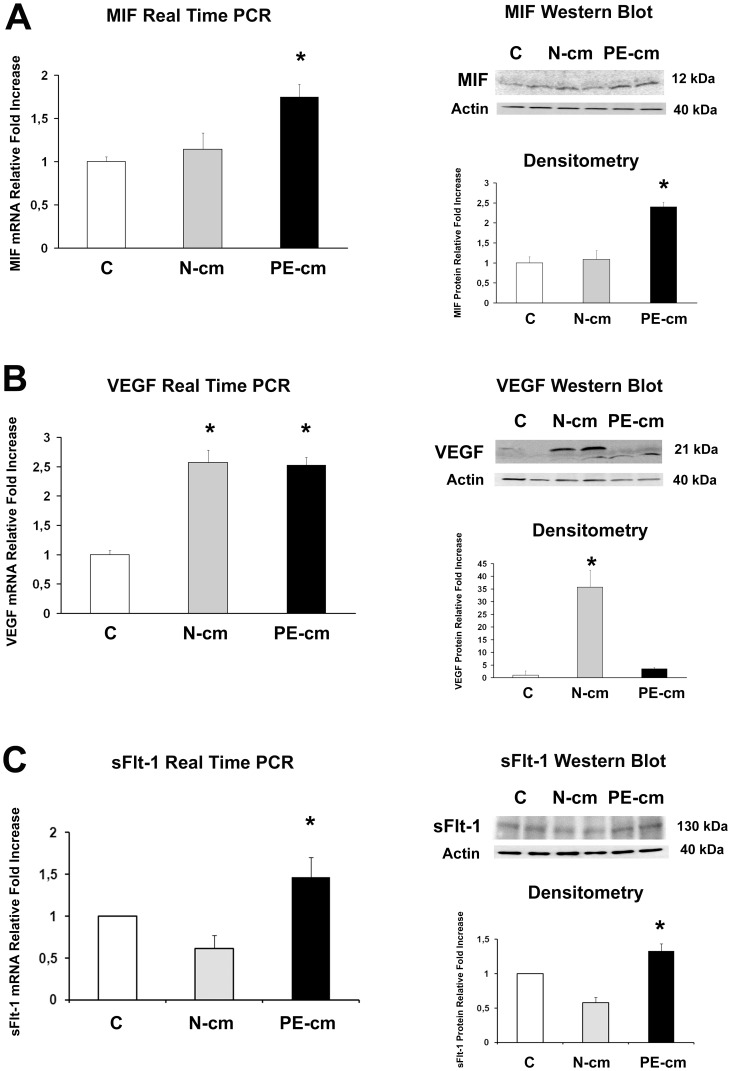
MIF, VEGF and sFlt-1 expression in physiological placental villous explants treated with culture media conditioned by normal or preeclamptic PDMSCs. MIF (A), VEGF (B) and sFlt-1 (C) mRNA (left panels) and protein (right panels) expression levels in physiological villous explants treated with unconditioned media [C] or media conditioned by normal [N-cm] and preeclamptic [PE-cm] PDMSCs as assessed by Real Time PCR and Western Blot analysis. Statistical significance (*) has been considered as p<0.05.

**Figure 5:**
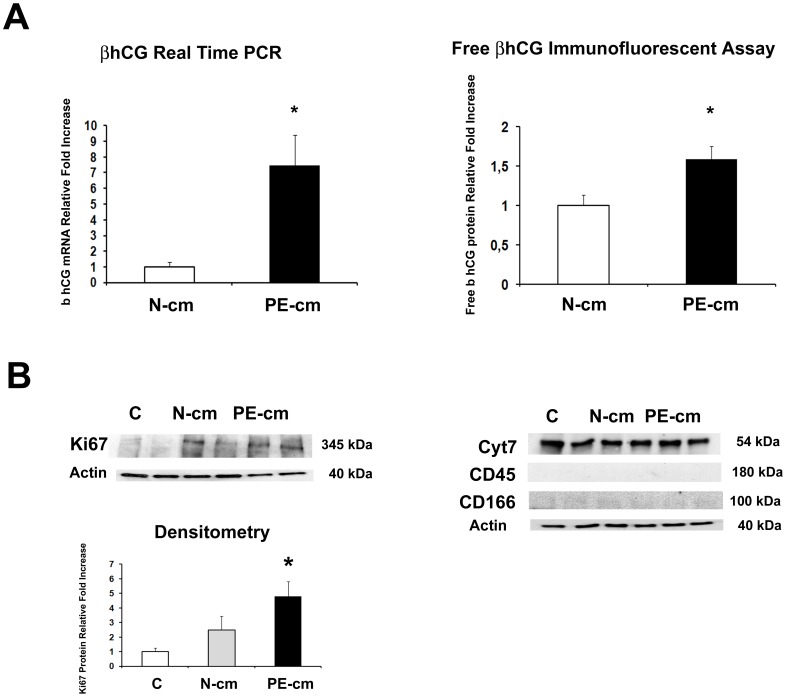
βhCG and Ki 67 expression in physiological placental villous explants treated by normal or PE PDMSCs conditioned media. A) βhCG mRNA (left panel) and free βhCG protein (right panel) levels in physiological villous explants treated with unconditioned [C], normal [N-cm] or preeclamptic [PE-cm] PDMSC conditioned media; B) Ki67 protein expression levels (left panel) in villous explants treated under the above mentioned experimental conditions. Figure B-right panel shows representative Western Blots for the characterization of villous explants cellular components: Cyt7 (trophoblast cell marker), CD45 (haematopoietic/endothelial cell marker) and CD166 (mesenchymal stromal cells marker). Statistical significance (*) has been considered as p<0.05.

Since we determined that preeclamptic PDMSCs released augmented concentrations of pro-inflammatory cytokines and chemotactic factors, we next investigated whether they were able to negatively influence inflammation and/or vasculogenesis on the placental tissue. To reach our goal, we treated term physiological chorionic villous explants with conditioned media from normal and PE PDMSCs. As markers of inflammation and vasculogenesis, we investigated MIF and VEGF expression. We found significantly increased MIF mRNA expression levels (p = 0.033, 1.75 Fold Increase) in PE PDMSCs CM explants relative to both untreated controls and explants treated with normal PDMSCs conditioned media (Figure 4A, left panel). Results were confirmed at the protein levels where we described significantly increased MIF protein levels (p = 0.027, 2.4 Fold Increase) in preeclamptic PDMSCs media explants relative to both untreated controls and explants treated with normal PDMSCs CM (Figure 4B, left upper and lower panels). VEGF mRNA expression was significantly increased in both PE and normal PDMSCs CM explants relative to untreated controls (p<0.01, 2.5 Fold Increase, Figure 4A, left panel). In stark contrast to mRNA data, only normal PDMSCs conditioned media was able to induce a significant VEGF protein accumulation (p<0.01, 35 Fold increase) relative to both untreated and PE PDMSCs CM explants (Figure 4B, right upper and lower panels). These findings were confirmed also by ELISA assay. Indeed, we found significantly increased VEGF protein concentration in normal PDMSCs CM-treated explants relative to both untreated controls (p<0.01, 1.4 Fold Increase) and PE-PDMSCs CM-treated explants (p<0.01, 1.6 Fold Increase) (Figure S3B, right panel). Anti-angiogenic sFlt-1 is the main responsible for VEGF down regulation during preeclampsia. Herein, we found significantly increased sFlt-1 mRNA expression in PE PDMSCs CM explants relative to both normal PDMSCs CM explants and untreated controls (p<0.001, 2.4 and 1.5 Fold Increase respectively; Figure 4C left panel). Data were confirmed at the protein levels, where we reported significantly increased sFlt-1 levels in explants treated by PE PDMSCs CM relative to both normal PDMSCs CM and untreated control explants (p = 0.003, 2.3 and 1.4 Fold Increase respectively; Figure 4C right upper and lower panels). Finally, we reported significantly increased expression of Ki67, marker of cell proliferation, in chorionic villous explants treated by PE PDMSCs media (p = 0.044, 4.8 Fold Increase) compared to normal PDMSCs CM explants and untreated controls (Figure 5B, left panel). The pro-inflammatory profile of PE-PDMSC CM explants was further confirmed by significantly increased TNF-α protein levels relative to both normal PDMSCs CM explants (p<0.01, 4.25 Fold Increase) and untreated controls (p = 0.014, 1.87 Fold Increase) as detected by ELISA assay (Figure S3B left panel).

Villous explants are representative of the whole placental tissue and composed of trophoblast, mesenchymal and endothelial cells. Thus, we investigated the contribution of these cell populations to the above mentioned results by determining the expression of Cyt7 (trophoblast marker), CD45 (haematopoietic/endothelial cell marker) and CD166 (MSCs marker) in our experimental model. Cyt7 was strongly expressed without differences in untreated, normal and PE PDMSCs CM explants (p<0.05, Figure 5B right upper panel). In contrast, CD45 and CD166 were barely detectable (Figure 5B right middle and lower panels), thus indicating that trophoblast cells were the main villous explants cellular component.

### Increased Release of Free βhCG by Villous Explants Treated with PE PDMSCs Conditioned Media

In order to further characterize the preeclamptic-like phenotype induced by PE PDMSCs conditioned media, we compared the amount of free βhCG, well recognized PE biomarker [28], [29], released by normal and PE PDMSCs CM explants. We found significantly increased βhCG mRNA levels in PE relative to normal PDMSCs CM explants (p = 0.01, 7.45 Fold Increase, Figure 5A left panel). Importantly, immunofluorescent assay confirmed mRNA results revealing significantly increased free βhCG levels (p = 0.03, 1.6 Fold Increase) in media from PE PDMSCs CM explants relative to controls (Figure 5A right panel).

## Discussion

Preeclampsia is a multi-factorial placenta-related syndrome [3] which anomalies originate early on during the first stages of placenta development. One of the most recognized theories describes the poor placentation typical of PE as the result of deficient mother-trophoblast immune acceptance [30]. This pathological condition will lead to diminished placenta perfusion and oxidative stress [31], [32], [33], [34], [35], increased trophoblast apoptosis and turnover [36], [37], [38], [39], [40], release of pro-inflammatory cytokines and syncitiotrophoblast debris into the maternal circulation that directly damage the endothelium [41] However, the precise pathogenic events leading to preeclampsia still remain unclear.

In the present study, we reported aberrant release of pro-inflammatory cytokines by mesenchymal stromal cells derived from preeclamptic placentae relative to normal PDMSCs. Moreover, we described a slow proliferation rate accompanied by increased senescence in PE-PDMSCs, suggesting a compromised self-renewal capacity of these cells resident in the villous stroma. Such a defect could significantly contribute to the aberrant villous architecture typical of PE placentae and it is in stark contrast with the well established hyper-proliferative phenotype of the neighboring PE trophoblast.

Importantly, we demonstrated for the first time to our knowledge that conditioned media from PE-PDMSCs induces a preeclamptic-like phenotype in normal placental villous explants. PE-PDMSC media promoted the expression of pro-inflammatory MIF and anti-angiogenic sFlt-1 in normal term villous explants while, at the protein level, it induced VEGF down regulation.

The discovery of such unique cells as MSCs resident in the placental tissues (basal plate, amnion and decidua) brought us to the hypothesis that MSCs themselves could contribute to the pathophysiology of placentation in virtue of their immune-regulatory and anti-inflammatory properties. MSCs exert an immunosuppressive effect on T-cells [11] and secrete paracrine factors that act on vasculogenesis and protect against ischemic injury [42]. Herein, we analyzed by cytokine array technology the expression of eighty different cytokines and growth factors in MSCs isolated from the chorionic villous portion of normal and PE placentae. We investigated this specific MSCs population because it localizes in the villous mesenchyme, thus being able to interact with the neighboring trophoblast, mesenchyme and endothelial cells.

Overall, we found higher expression of inflammatory molecules in media conditioned by PE relative to normal PDMSCs. In particular, preeclamptic PDMSCs released considerably elevated levels of IL-8, TGF-β2, TIMP-2, Osteoprotegerin, MCP-1, Osteopontin and, to a lesser extent, IL-6, TIMP-1, ENA-78, VEGF, Angiogenin, IGFBP-4, BDNF, LIF, IP10, PDGF-BB, MCP-3, TARC, TNF-α, Leptin and PlGF.

Hwang and colleagues recently characterized cytokine production profiles of MSCs isolated from amnion and decidua of healthy versus preeclamptic placentae [43]. Interestingly, they didn’t find differences in cytokines expression between normal and PE amnion-derived MSCs [43] while they reported higher levels of SDF-1 and no differences in MCP-1 expression in normal relative to PE decidual MSCs. Their results differ from our data most likely because we analyzed a different MSCs population isolated from the placental villi instead of the amnion or the decidua. In PE-PDMSCs media, we found increased levels of MCP-1 and MCP-3, type I inflammatory chemokines able to recruit monocytes, memory T cells, eosinophils and dendritic cells [44], [45], [46], [47]. During physiological early pregnancy, MCP-1 is produced by first trimester extravillous trophoblast cells following TNF stimulation [48], whereas MCP-3 is down-regulated through peri- and post-implantation stages to let the blastocyst invade and get acquainted to the uterine environment [49]. The aberrant release of Th1 cytokines (cell-mediated immune response) MCP-1 and/or MCP-3 by PE-PDMSCs is indicative of a failure in the Th2 (humoral immunity) switch typical of physiological pregnancies, necessary for the correct placental immune-acceptance by the maternal system.

In line with our results, Szarka and colleagues [50] reported increased levels of pro-inflammatory IL-8, IL-6, IP10, TNF-α and MCP-1 in serum from preeclamptic patients. This cytokines profile was indicated as an hallmark of abnormal pro-inflammatory systemic environment [50]. The increased release of these molecules by PE-PDMSCs could have important pathological implications. IL-8 is a potent chemoattractant for neutrophils while IL-6 has a key role in the acute-phase response [51]. IP-10 is a pro-inflammatory and anti-angiogenic cytokine that has been proposed to link inflammation and anti-angiogenesis in preeclampsia [52]. TNF-α is a type I cytokine promoter of cellular-mediated immune response that was previously reported as over-expressed in PE placentae and maternal serum [53], [54]. TNF-α inhibits trophoblast cell mobility *in vitro* [55] and it reduces trophoblast invasive capacity [56], [57], [58]. Our data suggest that PE-PDMSCs could directly affect trophoblast invasivity via TNF-α release. Moreover, TNF-α promotes the expression of several inflammatory molecules, thus inducing a vicious pro-inflammatory loop detrimental for pregnancy physiology.

Other molecules abundantly over-expressed in PE-PDMSCs media are TGF-β2, TIMP-2, Osteoprotegerin and Osteopontin. Transforming Growth Factor-βs have been indicated as main regulators of trophoblast invasion [59], [60]. Moreover, TGF-β1 [61], [62], TGF-β2 [63] and TGF-β3 [60] have been found increased in the maternal circulation and placental tissue of PE and FGR pregnancies, suggesting a role for TGF-βs in the trophoblast dysfunction typical of placenta-related pathologies. Herein, we found elevated levels of TGF-β2 and, to a lesser extent, TGF-β1 and TGF-β3 in the conditioned media of PE-PDMSCs, thus indicating a contribution of mesenchymal TGF-βs, TGF-β2 in particular, in PE dysfunction.

TIMPs are matrix metalloproteinase inhibitors that suppress proliferation of endothelial cells, thus exerting an anti-angiogenic activity. TIMP-2 has a key role during pregnancy in maintaining the homeostasis of trophoblast invasivity. Thus, TIMP-2 expression is low during the first stages of pregnancy, to increase with advancing gestation [64]. In accordance to our results, TIMP-2 immunoreactivity was previously found increased in syncitiotrophoblast and mesenchymal cells of PE placentae [65]. Data obtained in COMT knockout mice model, suggested that overproduction of both TIMP-2 and TGF-β3 may result in shallow utero-placental circulation and in the onset of a preeclamptic-like phenotype [65], [66].

Osteoprotegerin (OPG) inhibit bone resorption, regulates vascular integrity [67], [68], stimulates proliferation, inflammation and fibrogenesis in vascular smooth muscle cells [69]. The human placenta is considered the main source of OPG during gestation [70]. A significant increase in serum OPG concentration was observed during pregnancy [71] and, according to our data obtained on PE PDMSCs, placental and circulating OPG levels further increase in preeclampsia [72]. Osteopontin (OPN) is involved in adhesion and signal transduction at the uterine-placental interface during implantation and placentation [73]. Importantly, OPN acts as a pro-inflammatory Th1 type cytokine *in vivo* [74] and preeclamptic patients with extensive endothelial injury are characterized by increased plasma levels of OPN [75]. Thus, the increased OPN production by PE-PDMSCs that we reported could cause and/or further contribute to the aberrant placental inflammation underlying preeclampsia.

PE-PDMSCs expressed also higher levels of pro-angiogenic growth factors VEGF, Angiogenin and PlGF. Besides being pivotal for placentation, these angiogenic molecules can be strong endothelial cell activators. VEGF role in PE pregnancy has been controversially discussed, as its levels in PE maternal serum have been reported as increased [76], [77], [78], decreased [79], [80], or even unchanged [81] Angiogenin expression in the human placenta increases during gestation [82] and it is further up-regulated in fetal growth restricted pregnancies relative to controls [83]. Placental Growth Factor (PlGF) expression is limited to the placental tissue where it promotes villous angiogenesis during early gestation. PlGF plasma levels and function are dramatically compromised in PE patients, resulting in endothelial injury and vascular permeability [22]. Indeed, increased PlGF expression by PE-PDMSCs could also indicate a compensatory mechanisms acted to induce new angiogenesis in the compromised PE placental vasculature.

Leptin, LIF, PDGF-BB and BDNF are as well augmented in PE-PDMSCs relative to control PDMSCs media. During pregnancy, these cytokines are involved in critical processes as placental invasion [84], trophoblast cell proliferation, fetal growth and development [84], [85]. Moreover, they stimulates cytokines production [84] and are directly implicated in inflammation [85], suggesting a pathophysiological role in preeclampsia. Leptin and LIF have been previously reported to be over-expressed at both placental and plasma levels in severe preeclamptic pregnancies [86], [87]. Leptin, in particular, could aggravate hypertension and endothelial damage by promoting catecholamine production [86]. On the other hand, PDGF-BB is a potent chemoattractor that drives mesenchymal cells migration [88], thus its increase in PE-PDMSCs media could be interpreted as an attempt to recruit MSCs and repair placental damage.

Taken together, our data clearly indicate that preeclamptic MSCs resident in the chorionic placental villi over-produce a repertoire of cytokines, chemokines and growth factors that could directly contribute to the anomalies of trophoblast development and placental angiogenesis and to the exacerbated inflammatory response typical of PE and FGR. These molecules, crucial for proper pregnancy outcome under normal conditions, when abnormally expressed are detrimental for placenta physiology. In line with our results, Waterman and colleagues described two different immune-modulating profiles in human MSCs isolated from bone marrow. They found pro-inflammatory Toll-like Receptor 4 (TLR4) primed MSCs, that expressed high levels of IL-8, IL-6 and TGF-β2 molecules, and immunosuppressive TLR3-primed MSCs, characterized by marked expression of IL-4. IL-10, RANTES and IP-10 [89]. We reported significantly increased levels of IL-8, IL-6 and TGF-β2 cytokines in preeclamptic placental MSCs relative to controls, thus indicating that PE PDMSCS resemble a pro-inflammatory TLR4-primed phenotype.

Therefore, we next investigated whether PE-PDMSCs could alter the physiological placental equilibrium. We treated normal chorionic villous explants with media conditioned by normal or PE PDMSCs and investigated the expression of MIF, VEGF, sFlt-1, Ki67 and βhCG molecules. MIF is a central regulator of inflammation and immune response [90] described in both villous and extravillous trophoblast in humans [91], [92]. Emerging evidences emphasize its importance in early pregnancy, embryonic development and preeclampsia [19], [91], [92], [93]. Herein, we found that normal villous explants treated with PE PDMSCs conditioned media over-expressed MIF at both gene and protein levels, while CM from normal PDMSCs did not affect physiological MIF production. Our group previously reported that MIF serum levels were significantly increased in women affected by severe PE [19], [93], thus entailing a role for MIF in the etiopathogenesis of PE. Importantly, TNF-α, beside being directly induced by MIF, promotes MIF production by a positive feedback [94]. Indeed, the high TNF-α levels secreted by PE PDMSCs could explain MIF mRNA and protein accumulation. This PE-PDMSCs induced alteration in MIF expression further confirm the contribution of pathological mesenchymal stromal cells to the pathogenesis of preeclampsia.

As discussed above, VEGF orchestrates angiogenesis and it is vital for early embryonic development [95]. VEGF is involved also in the pathogenesis of preeclampsia. The preeclamptic placenta over-expresses VEGF in response to the hypoxic environment [21], [96], but free and biologically active VEGF protein is sequestered and down-regulated by sFlt-1, soluble variant of VEGF receptor 1 released in excess by the PE placenta [22], [23], [24], [25]. In the present study, both normal and preeclamptic PDMSCs’s media promoted VEGF mRNA expression compared to untreated chorionic villous explants, while only PE PDMSCs media induced sFlt-1 mRNA accumulation. In accordance to mRNA data, normal PDMSCs’s media treatment was accompanied by VEGF protein accumulation, thus confirming the well documented pro-angiogenic activity of mesenchymal stromal cells [42]. In stark contrast, treatment with PE PDMSCs’s media resulted not in VEGF but in sFlt-1 protein increase. Our results resemble the *in vivo* condition typical of preeclampsia, where the VEGF placental mRNA increase induced by the hypoxic and pro-inflammatory environment is antagonized by the over-expression of anti-angiogenic sFlt-1.

Moreover, preeclamptic placentae are characterized by immature hyper-proliferative trophoblast phenotype. Thus, we investigated Ki67 expression in villous explants treated by normal or PE PDMSCs conditioned media. We reported significant Ki67increase in PE-PDMSCs CM explants, indicating the induction of an hypertrophic PE-like status by preeclamptic PDMSCs.

As a further confirmation of the PE-like environment provoked by PE-PDMSCs, we reported augmented release of free βhCG by normal villous explants treated with preeclamptic PDMSCs media. hCG is normally secreted by trophoblast cells to maintain maternal vascular supply during pregnancy and its increased circulating levels have been associated with a dramatically higher risk of developing preeclampsia [28], [29].

We are currently investigating whether normal placental MSCs are able to revert the pathological inflammatory conditions typical of PE placentae.

In conclusion, our data clearly depict chorionic placental mesenchymal stromal cells as central players in placental physiopathology, thus opening to new intriguing perspectives for the treatment of human placental-related disorders as preeclampsia and fetal growth restriction.

## Supporting Information

Figure S1 **Placenta-derived Mesenchymal Stromal Cells Characterization.** A) Representative phenotype of human chorionic PDMSCs at passage 5 as assessed by flow cytometry. All cells were positive for CD166, CD105, CD90, CD73 and negative for HLA II, CD34, CD133, CD20, CD326, CD31, CD45 and CD14, thus displaying proper mesenchymal profile and no contamination from epithelial, hematopoietic, immune or endothelial cells. B) Representative Oct4 and Nanog PCR analysis in control and PE PDMSCs. All cell lines expressed both gene markers of stemness.
(TIF)

Figure S2 **Gene expression levels of key differentially expressed molecules detected by Cytokine Array in normal (C) and preeclamptic (PE) PDMSCs.** MIF (A), VEGF (B), TNF-α (C), IL-8 (D) and IL-6 (E) mRNA expression levels in normal and PE-PDMSCs as assessed by Real Time PCR. Statistical significance (*) has been considered as p<0.05.
(TIF)

Figure S3 **TNF-α and VEGF protein expression in Normal and PE-PDMSCs Conditioned Media and in physiological placental villous explants treated by normal or PE PDMSCs CM as detected by ELISA Assay.** (A) TNF-α (left panel) and VEGF (right panel) protein levels in media conditioned by Normal [N-cm] or preeclamptic [PE-cm] PDMSCs. (B) TNF-α (left panel) and VEGF (right panel) protein levels in untreated control explants [C] and explants treated by normal [N-cm] and preeclamptic [PE-cm] PDMSCs conditioned medium. Results are expressed as means ± SE. Statistical significance (*) has been considered as p<0.05.
(TIF)

Table S1 **Cytokines Expression in Normal vs Preeclamptic PDMSCs Conditioned Media.**
(DOC)

## References

[1] Chesley LC (1985) Diagnosis of preeclampsia. Obstet Gynecol 65: 423–425. 3883267

[2] ACOG practice bulletin. Diagnosis and management of preeclampsia and eclampsia. Number 33, January 2002. Obstet Gynecol 99: 159–167. 16175681 10.1016/s0029-7844(01)01747-1

[3] Redman CW, Sargent IL (2005) Latest advances in understanding preeclampsia. Science 308: 1592–1594. 15947178 10.1126/science.1111726

[4] Roberts JM, Gammill HS (2005) Preeclampsia: recent insights. Hypertension 46: 1243–1249. 16230510 10.1161/01.HYP.0000188408.49896.c5

[5] Cunningham FG, Lindheimer MD (1992) Hypertension in pregnancy. N Engl J Med 326: 927–932. 1542342 10.1056/NEJM199204023261405

[6] Pijnenborg R, Vercruysse L, Verbist L, Van Assche FA (1998) Interaction of interstitial trophoblast with placental bed capillaries and venules of normotensive and pre-eclamptic pregnancies. Placenta 19: 569–575. 9859859 10.1016/s0143-4004(98)90016-9

[7] Lockwood CJ, Yen CF, Basar M, Kayisli UA, Martel M, et al (2008) Preeclampsia-related inflammatory cytokines regulate interleukin-6 expression in human decidual cells. Am J Pathol 172: 1571–1579. 18467705 10.2353/ajpath.2008.070629 PMC2408417

[8] Sibai B, Dekker G, Kupferminc M (2005) Pre-eclampsia. Lancet 365: 785–799. 15733721 10.1016/S0140-6736(05)17987-2

[9] Gammill HS, Roberts JM (2007) Emerging concepts in preeclampsia investigation. Front Biosci 12: 2403–2411. 17127250 10.2741/2242

[10] Fukuchi Y, Nakajima H, Sugiyama D, Hirose I, Kitamura T, et al (2004) Human placenta-derived cells have mesenchymal stem/progenitor cell potential. Stem Cells 22: 649–658. 15342929 10.1634/stemcells.22-5-649

[11] Li C, Zhang W, Jiang X, Mao N (2007) Human-placenta-derived mesenchymal stem cells inhibit proliferation and function of allogeneic immune cells. Cell Tissue Res 330: 437–446. 17899199 10.1007/s00441-007-0504-5

[12] Chang CJ, Yen ML, Chen YC, Chien CC, Huang HI, et al (2006) Placenta-derived multipotent cells exhibit immunosuppressive properties that are enhanced in the presence of interferon-gamma. Stem Cells 24: 2466–2477. 17071860 10.1634/stemcells.2006-0071

[13] Parolini O, Alviano F, Bergwerf I, Boraschi D, De Bari C, et al. Toward cell therapy using placenta-derived cells: disease mechanisms, cell biology, preclinical studies, and regulatory aspects at the round table. Stem Cells Dev 19: 143–154. 19947828 10.1089/scd.2009.0404

[14] Croy BA, Chantakru S, Esadeg S, Ashkar AA, Wei Q (2002) Decidual natural killer cells: key regulators of placental development (a review). J Reprod Immunol 57: 151–168. 12385840 10.1016/s0165-0378(02)00005-0

[15] Boyson JE, Rybalov B, Koopman LA, Exley M, Balk SP, et al (2002) CD1d and invariant NKT cells at the human maternal-fetal interface. Proc Natl Acad Sci U S A 99: 13741–13746. 12368486 10.1073/pnas.162491699 PMC129762

[16] Guimond MJ, Wang B, Croy BA (1998) Engraftment of bone marrow from severe combined immunodeficient (SCID) mice reverses the reproductive deficits in natural killer cell-deficient tg epsilon 26 mice. J Exp Med 187: 217–223. 9432979 10.1084/jem.187.2.217 PMC2212103

[17] Croy BA, Ashkar AA, Minhas K, Greenwood JD (2000) Can murine uterine natural killer cells give insights into the pathogenesis of preeclampsia? J Soc Gynecol Investig 7: 12–20. 10.1016/s1071-5576(99)00049-0 10732312

[18] Reister F, Frank HG, Heyl W, Kosanke G, Huppertz B, et al (1999) The distribution of macrophages in spiral arteries of the placental bed in pre-eclampsia differs from that in healthy patients. Placenta 20: 229–233. 10195746 10.1053/plac.1998.0373

[19] Cardaropoli S, Paulesu L, Romagnoli R, Ietta F, Marzioni D, et al (2011) Macrophage migration inhibitory factor in fetoplacental tissues from preeclamptic pregnancies with or without fetal growth restriction. Clin Dev Immunol 2012: 639342. 22007254 10.1155/2012/639342 PMC3189467

[20] Ahmed A, Li XF, Dunk C, Whittle MJ, Rushton DI, et al (1995) Colocalisation of vascular endothelial growth factor and its Flt-1 receptor in human placenta. Growth Factors 12: 235–243. 8619929 10.3109/08977199509036883

[21] Chung JY, Song Y, Wang Y, Magness RR, Zheng J (2004) Differential expression of vascular endothelial growth factor (VEGF), endocrine gland derived-VEGF, and VEGF receptors in human placentas from normal and preeclamptic pregnancies. J Clin Endocrinol Metab 89: 2484–2490. 15126581 10.1210/jc.2003-031580 PMC3282114

[22] Levine RJ, Maynard SE, Qian C, Lim KH, England LJ, et al (2004) Circulating angiogenic factors and the risk of preeclampsia. N Engl J Med 350: 672–683. 14764923 10.1056/NEJMoa031884

[23] Nevo O, Soleymanlou N, Wu Y, Xu J, Kingdom J, et al (2006) Increased expression of sFlt-1 in in vivo and in vitro models of human placental hypoxia is mediated by HIF-1. Am J Physiol Regul Integr Comp Physiol 291: R1085–1093. 16627691 10.1152/ajpregu.00794.2005 PMC6428068

[24] Koga K, Osuga Y, Yoshino O, Hirota Y, Ruimeng X, et al (2003) Elevated serum soluble vascular endothelial growth factor receptor 1 (sVEGFR-1) levels in women with preeclampsia. J Clin Endocrinol Metab 88: 2348–2351. 12727995 10.1210/jc.2002-021942

[25] Ahmad S, Ahmed A (2004) Elevated placental soluble vascular endothelial growth factor receptor-1 inhibits angiogenesis in preeclampsia. Circ Res 95: 884–891. 15472115 10.1161/01.RES.0000147365.86159.f5

[26] Brooke G, Rossetti T, Pelekanos R, Ilic N, Murray P, et al (2009) Manufacturing of human placenta-derived mesenchymal stem cells for clinical trials. Br J Haematol 144: 571–579. 19077161 10.1111/j.1365-2141.2008.07492.x

[27] Livak KJ, Schmittgen TD (2001) Analysis of relative gene expression data using real-time quantitative PCR and the 2(-Delta Delta C(T)) Method. Methods 25: 402–408. 11846609 10.1006/meth.2001.1262

[28] Olsen RN, Woelkers D, Dunsmoor-Su R, Lacoursiere DY (2012) Abnormal second-trimester serum analytes are more predictive of preterm preeclampsia. Am J Obstet Gynecol 207: 228 e221–227. 10.1016/j.ajog.2012.06.006 22818876

[29] Norris W, Nevers T, Sharma S, Kalkunte S (2011) Review: hCG, preeclampsia and regulatory T cells. Placenta 32 Suppl 2S182–185. 21295851 10.1016/j.placenta.2011.01.009 PMC3061407

[30] King A, Hiby SE, Gardner L, Joseph S, Bowen JM, et al.. (2000) Recognition of trophoblast HLA class I molecules by decidual NK cell receptors–a review. Placenta 21 Suppl A: S81–85. 10.1053/plac.1999.0520 10831129

[31] Roberts JM, Hubel CA (1999) Is oxidative stress the link in the two-stage model of pre-eclampsia? Lancet 354: 788–789. 10485715 10.1016/S0140-6736(99)80002-6

[32] Hubel CA (1999) Oxidative stress in the pathogenesis of preeclampsia. Proc Soc Exp Biol Med 222: 222–235. 10601881 10.1177/153537029922200305

[33] Shibata E, Ejima K, Nanri H, Toki N, Koyama C, et al (2001) Enhanced protein levels of protein thiol/disulphide oxidoreductases in placentae from pre-eclamptic subjects. Placenta 22: 566–572. 11440545 10.1053/plac.2001.0693

[34] Myatt L, Cui X (2004) Oxidative stress in the placenta. Histochem Cell Biol 122: 369–382. 15248072 10.1007/s00418-004-0677-x

[35] Burton GJ, Yung HW, Cindrova-Davies T, Charnock-Jones DS (2009) Placental endoplasmic reticulum stress and oxidative stress in the pathophysiology of unexplained intrauterine growth restriction and early onset preeclampsia. Placenta 30 Suppl A: S43–48. 10.1016/j.placenta.2008.11.003 PMC2684656 19081132

[36] Allaire AD, Ballenger KA, Wells SR, McMahon MJ, Lessey BA (2000) Placental apoptosis in preeclampsia. Obstet Gynecol 96: 271–276. 10908776 10.1016/s0029-7844(00)00895-4

[37] Ishihara N, Matsuo H, Murakoshi H, Laoag-Fernandez JB, Samoto T, et al (2002) Increased apoptosis in the syncytiotrophoblast in human term placentas complicated by either preeclampsia or intrauterine growth retardation. Am J Obstet Gynecol 186: 158–166. 11810103 10.1067/mob.2002.119176

[38] Leung DN, Smith SC, To KF, Sahota DS, Baker PN (2001) Increased placental apoptosis in pregnancies complicated by preeclampsia. Am J Obstet Gynecol 184: 1249–1250. 11349196 10.1067/mob.2001.112906

[39] Crocker IP, Cooper S, Ong SC, Baker PN (2003) Differences in apoptotic susceptibility of cytotrophoblasts and syncytiotrophoblasts in normal pregnancy to those complicated with preeclampsia and intrauterine growth restriction. Am J Pathol 162: 637–643. 12547721 10.1016/S0002-9440(10)63857-6 PMC1851173

[40] Soleymanlou N, Wu Y, Wang JX, Todros T, Ietta F, et al (2005) A novel Mtd splice isoform is responsible for trophoblast cell death in pre-eclampsia. Cell Death Differ 12: 441–452. 15775999 10.1038/sj.cdd.4401593

[41] Smarason AK, Sargent IL, Starkey PM, Redman CW (1993) The effect of placental syncytiotrophoblast microvillous membranes from normal and pre-eclamptic women on the growth of endothelial cells in vitro. Br J Obstet Gynaecol 100: 943–949. 8217980 10.1111/j.1471-0528.1993.tb15114.x

[42] Caplan AI, Dennis JE (2006) Mesenchymal stem cells as trophic mediators. J Cell Biochem 98: 1076–1084. 16619257 10.1002/jcb.20886

[43] Hwang JH, Lee MJ, Seok OS, Paek YC, Cho GJ, et al (2011) Cytokine expression in placenta-derived mesenchymal stem cells in patients with pre-eclampsia and normal pregnancies. Cytokine 49: 95–101. 10.1016/j.cyto.2009.08.013 19819721

[44] Carr MW, Roth SJ, Luther E, Rose SS, Springer TA (1994) Monocyte chemoattractant protein 1 acts as a T-lymphocyte chemoattractant. Proc Natl Acad Sci U S A 91: 3652–3656. 8170963 10.1073/pnas.91.9.3652 PMC43639

[45] Xu LL, Warren MK, Rose WL, Gong W, Wang JM (1996) Human recombinant monocyte chemotactic protein and other C-C chemokines bind and induce directional migration of dendritic cells in vitro. J Leukoc Biol 60: 365–371. 8830793 10.1002/jlb.60.3.365

[46] Sozzani S, Zhou D, Locati M, Rieppi M, Proost P, et al (1994) Receptors and transduction pathways for monocyte chemotactic protein-2 and monocyte chemotactic protein-3. Similarities and differences with MCP-1. J Immunol 152: 3615–3622. 8144937

[47] Dahinden CA, Geiser T, Brunner T, von Tscharner V, Caput D, et al (1994) Monocyte chemotactic protein 3 is a most effective basophil- and eosinophil-activating chemokine. J Exp Med 179: 751–756. 7507512 10.1084/jem.179.2.751 PMC2191381

[48] Renaud SJ, Sullivan R, Graham CH (2009) Tumour necrosis factor alpha stimulates the production of monocyte chemoattractants by extravillous trophoblast cells via differential activation of MAPK pathways. Placenta 30: 313–319. 19201463 10.1016/j.placenta.2009.01.001

[49] Nautiyal J, Kumar PG, Laloraya M (2004) Mifepristone (Ru486) antagonizes monocyte chemotactic protein-3 down-regulation at early mouse pregnancy revealing immunomodulatory events in Ru486 induced abortion. Am J Reprod Immunol 52: 8–18. 15214937 10.1111/j.1600-0897.2004.00176.x

[50] Szarka A, Rigo J, Jr., Lazar L, Beko G, Molvarec A Circulating cytokines, chemokines and adhesion molecules in normal pregnancy and preeclampsia determined by multiplex suspension array. BMC Immunol 11: 59. 21126355 10.1186/1471-2172-11-59 PMC3014878

[51] Gabay C, Kushner I (1999) Acute-phase proteins and other systemic responses to inflammation. N Engl J Med 340: 448–454. 9971870 10.1056/NEJM199902113400607

[52] Gotsch F, Romero R, Friel L, Kusanovic JP, Espinoza J, et al (2007) CXCL10/IP-10: a missing link between inflammation and anti-angiogenesis in preeclampsia? J Matern Fetal Neonatal Med 20: 777–792. 17943641 10.1080/14767050701483298 PMC2396489

[53] Wang Y, Walsh SW (1996) TNF alpha concentrations and mRNA expression are increased in preeclamptic placentas. J Reprod Immunol 32: 157–169. 9023819 10.1016/s0165-0378(96)00998-9

[54] Beckmann I, Efraim SB, Vervoort M, Visser W, Wallenburg HC (2004) Tumor necrosis factor-alpha in whole blood cultures of preeclamptic patients and healthy pregnant and nonpregnant women. Hypertens Pregnancy 23: 319–329. 15617632 10.1081/PRG-200030334

[55] Todt JC, Yang Y, Lei J, Lauria MR, Sorokin Y, et al (1996) Effects of tumor necrosis factor-alpha on human trophoblast cell adhesion and motility. Am J Reprod Immunol 36: 65–71. 8862248 10.1111/j.1600-0897.1996.tb00141.x

[56] Bauer S, Pollheimer J, Hartmann J, Husslein P, Aplin JD, et al (2004) Tumor necrosis factor-alpha inhibits trophoblast migration through elevation of plasminogen activator inhibitor-1 in first-trimester villous explant cultures. J Clin Endocrinol Metab 89: 812–822. 14764800 10.1210/jc.2003-031351

[57] Renaud SJ, Postovit LM, Macdonald-Goodfellow SK, McDonald GT, Caldwell JD, et al (2005) Activated macrophages inhibit human cytotrophoblast invasiveness in vitro. Biol Reprod 73: 237–243. 15800179 10.1095/biolreprod.104.038000

[58] Xu B, Nakhla S, Makris A, Hennessy A TNF-alpha inhibits trophoblast integration into endothelial cellular networks. Placenta 32: 241–246. 21215448 10.1016/j.placenta.2010.12.005

[59] Simpson H, Robson SC, Bulmer JN, Barber A, Lyall F (2002) Transforming growth factor beta expression in human placenta and placental bed during early pregnancy. Placenta 23: 44–58. 11869091 10.1053/plac.2001.0746

[60] Caniggia I, Grisaru-Gravnosky S, Kuliszewsky M, Post M, Lye SJ (1999) Inhibition of TGF-beta 3 restores the invasive capability of extravillous trophoblasts in preeclamptic pregnancies. J Clin Invest 103: 1641–1650. 10377170 10.1172/JCI6380 PMC408387

[61] Todros T, Marzioni D, Lorenzi T, Piccoli E, Capparuccia L, et al (2007) Evidence for a role of TGF-beta1 in the expression and regulation of alpha-SMA in fetal growth restricted placentae. Placenta 28: 1123–1132. 17664003 10.1016/j.placenta.2007.06.003

[62] Djurovic S, Schjetlein R, Wisloff F, Haugen G, Husby H, et al (1997) Plasma concentrations of Lp(a) lipoprotein and TGF-beta1 are altered in preeclampsia. Clin Genet 52: 371–376. 9520129 10.1111/j.1399-0004.1997.tb04356.x

[63] Shaarawy M, El Meleigy M, Rasheed K (2001) Maternal serum transforming growth factor beta-2 in preeclampsia and eclampsia, a potential biomarker for the assessment of disease severity and fetal outcome. J Soc Gynecol Investig 8: 27–31. 11223354

[64] Pang ZJ, Zhou JG, Huang LP (2008) Interleukin-10 may participate in regulating trophoblast invasion in human placentae throughout gestation. Am J Reprod Immunol 60: 19–25. 18593434 10.1111/j.1600-0897.2008.00586.x

[65] Lee SB, Wong AP, Kanasaki K, Xu Y, Shenoy VK, et al. Preeclampsia: 2-methoxyestradiol induces cytotrophoblast invasion and vascular development specifically under hypoxic conditions. Am J Pathol 176: 710–720. 20075204 10.2353/ajpath.2010.090513 PMC2808078

[66] Kanasaki K, Palmsten K, Sugimoto H, Ahmad S, Hamano Y, et al (2008) Deficiency in catechol-O-methyltransferase and 2-methoxyoestradiol is associated with pre-eclampsia. Nature 453: 1117–1121. 18469803 10.1038/nature06951

[67] Kiechl S, Schett G, Wenning G, Redlich K, Oberhollenzer M, et al (2004) Osteoprotegerin is a risk factor for progressive atherosclerosis and cardiovascular disease. Circulation 109: 2175–2180. 15117849 10.1161/01.CIR.0000127957.43874.BB

[68] Van Campenhout A, Golledge J (2009) Osteoprotegerin, vascular calcification and atherosclerosis. Atherosclerosis 204: 321–329. 19007931 10.1016/j.atherosclerosis.2008.09.033 PMC2729052

[69] Toffoli B, Pickering RJ, Tsorotes D, Wang B, Bernardi S, et al. Osteoprotegerin promotes vascular fibrosis via a TGF-beta1 autocrine loop. Atherosclerosis 218: 61–68. 21679949 10.1016/j.atherosclerosis.2011.05.019

[70] Simonet WS, Lacey DL, Dunstan CR, Kelley M, Chang MS, et al (1997) Osteoprotegerin: a novel secreted protein involved in the regulation of bone density. Cell 89: 309–319. 9108485 10.1016/s0092-8674(00)80209-3

[71] Naylor KE, Rogers A, Fraser RB, Hall V, Eastell R, et al (2003) Serum osteoprotegerin as a determinant of bone metabolism in a longitudinal study of human pregnancy and lactation. J Clin Endocrinol Metab 88: 5361–5365. 14602774 10.1210/jc.2003-030486

[72] Vitoratos N, Lambrinoudaki I, Rizos D, Armeni E, Alexandrou A, et al. Maternal circulating osteoprotegerin and soluble RANKL in pre-eclamptic women. Eur J Obstet Gynecol Reprod Biol 154: 141–145. 21074311 10.1016/j.ejogrb.2010.10.009

[73] Johnson GA, Burghardt RC, Bazer FW, Spencer TE (2003) Osteopontin: roles in implantation and placentation. Biol Reprod 69: 1458–1471. 12890718 10.1095/biolreprod.103.020651

[74] Ashkar S, Weber GF, Panoutsakopoulou V, Sanchirico ME, Jansson M, et al (2000) Eta-1 (osteopontin): an early component of type-1 (cell-mediated) immunity. Science 287: 860–864. 10657301 10.1126/science.287.5454.860

[75] Stenczer B, Rigo J Jr, Prohaszka Z, Derzsy Z, Lazar L, et al (2012) Plasma osteopontin concentrations in preeclampsia - is there an association with endothelial injury? Clin Chem Lab Med 48: 181–187. 10.1515/CCLM.2010.042 19943814

[76] Baker PN, Krasnow J, Roberts JM, Yeo KT (1995) Elevated serum levels of vascular endothelial growth factor in patients with preeclampsia. Obstet Gynecol 86: 815–821. 7566855 10.1016/0029-7844(95)00259-T

[77] Hayman R, Brockelsby J, Kenny L, Baker P (1999) Preeclampsia: the endothelium, circulating factor(s) and vascular endothelial growth factor. J Soc Gynecol Investig 6: 3–10. 10065419

[78] Sharkey AM, Cooper JC, Balmforth JR, McLaren J, Clark DE, et al (1996) Maternal plasma levels of vascular endothelial growth factor in normotensive pregnancies and in pregnancies complicated by pre-eclampsia. Eur J Clin Invest 26: 1182–1185. 9013097 10.1046/j.1365-2362.1996.830605.x

[79] Lyall F, Greer IA, Boswell F, Fleming R (1997) Suppression of serum vascular endothelial growth factor immunoreactivity in normal pregnancy and in pre-eclampsia. Br J Obstet Gynaecol 104: 223–228. 9070144 10.1111/j.1471-0528.1997.tb11050.x

[80] Livingston JC, Chin R, Haddad B, McKinney ET, Ahokas R, et al (2000) Reductions of vascular endothelial growth factor and placental growth factor concentrations in severe preeclampsia. Am J Obstet Gynecol 183: 1554–1557. 11120527 10.1067/mob.2000.108022

[81] Hefler L, Obermair A, Husslein P, Kainz C, Tempfer C (2000) Vascular endothelial growth factor serum levels in pregnancy and preeclampsia. Acta Obstet Gynecol Scand 79: 77–78. 10646821

[82] Rajashekhar G, Loganath A, Roy AC, Wong YC (2002) Expression and localization of angiogenin in placenta: enhanced levels at term over first trimester villi. Mol Reprod Dev 62: 159–166. 11984825 10.1002/mrd.10116

[83] Rajashekhar G, Loganath A, Roy AC, Wong YC (2003) Over-expression and secretion of angiogenin in intrauterine growth retardation placenta. Mol Reprod Dev 64: 397–404. 12589651 10.1002/mrd.10229

[84] Maymo JL, Perez AP, Gambino Y, Calvo JC, Sanchez-Margalet V, et al. Review: Leptin gene expression in the placenta–regulation of a key hormone in trophoblast proliferation and survival. Placenta 32 Suppl 2S146–153. 21303721 10.1016/j.placenta.2011.01.004

[85] Gearing DP (1993) The leukemia inhibitory factor and its receptor. Adv Immunol 53: 31–58. 8512038 10.1016/s0065-2776(08)60497-6

[86] Sagawa N, Yura S, Itoh H, Kakui K, Takemura M, et al (2002) Possible role of placental leptin in pregnancy: a review. Endocrine 19: 65–71. 12583603 10.1385/ENDO:19:1:65

[87] Benian A, Uzun H, Aydin S, Albayrak M, Uludag S, et al (2008) Placental stem cell markers in pre-eclampsia. Int J Gynaecol Obstet 100: 228–233. 18048043 10.1016/j.ijgo.2007.09.023

[88] Nedeau AE, Bauer RJ, Gallagher K, Chen H, Liu ZJ, et al (2008) A CXCL5- and bFGF-dependent effect of PDGF-B-activated fibroblasts in promoting trafficking and differentiation of bone marrow-derived mesenchymal stem cells. Exp Cell Res 314: 2176–2186. 18570917 10.1016/j.yexcr.2008.04.007 PMC2638214

[89] Waterman RS, Tomchuck SL, Henkle SL, Betancourt AM A new mesenchymal stem cell (MSC) paradigm: polarization into a pro-inflammatory MSC1 or an Immunosuppressive MSC2 phenotype. PLoS One 5: e10088. 10.1371/journal.pone.0010088 PMC2859930 20436665

[90] Nishihira J (2000) Macrophage migration inhibitory factor (MIF): its essential role in the immune system and cell growth. J Interferon Cytokine Res 20: 751–762. 11032394 10.1089/10799900050151012

[91] Arcuri F, Cintorino M, Vatti R, Carducci A, Liberatori S, et al (1999) Expression of macrophage migration inhibitory factor transcript and protein by first-trimester human trophoblasts. Biol Reprod 60: 1299–1303. 10330084 10.1095/biolreprod60.6.1299

[92] Arcuri F, Ricci C, Ietta F, Cintorino M, Tripodi SA, et al (2001) Macrophage migration inhibitory factor in the human endometrium: expression and localization during the menstrual cycle and early pregnancy. Biol Reprod 64: 1200–1205. 11259268 10.1095/biolreprod64.4.1200

[93] Todros T, Bontempo S, Piccoli E, Ietta F, Romagnoli R, et al (2005) Increased levels of macrophage migration inhibitory factor (MIF) in preeclampsia. Eur J Obstet Gynecol Reprod Biol 123: 162–166. 15894418 10.1016/j.ejogrb.2005.03.014

[94] Hirokawa J, Sakaue S, Furuya Y, Ishii J, Hasegawa A, et al (1998) Tumor necrosis factor-alpha regulates the gene expression of macrophage migration inhibitory factor through tyrosine kinase-dependent pathway in 3T3-L1 adipocytes. J Biochem 123: 733–739. 9538268 10.1093/oxfordjournals.jbchem.a021998

[95] Jelkmann W (2001) Pitfalls in the measurement of circulating vascular endothelial growth factor. Clin Chem 47: 617–623. 11274009

[96] Li H, Gu B, Zhang Y, Lewis DF, Wang Y (2005) Hypoxia-induced increase in soluble Flt-1 production correlates with enhanced oxidative stress in trophoblast cells from the human placenta. Placenta 26: 210–217. 15708122 10.1016/j.placenta.2004.05.004

