# Supplementary figures and images for: Pro-Inflammatory Profile of Preeclamptic Placental Mesenchymal Stromal Cells: New Insights into the Etiopathogenesis of Preeclampsia
Source: PLoS One. 2013 Mar 19;8(3):e59403. doi: 10.1371/journal.pone.0059403 (PMC3602067; doi:10.1371/journal.pone.0059403)

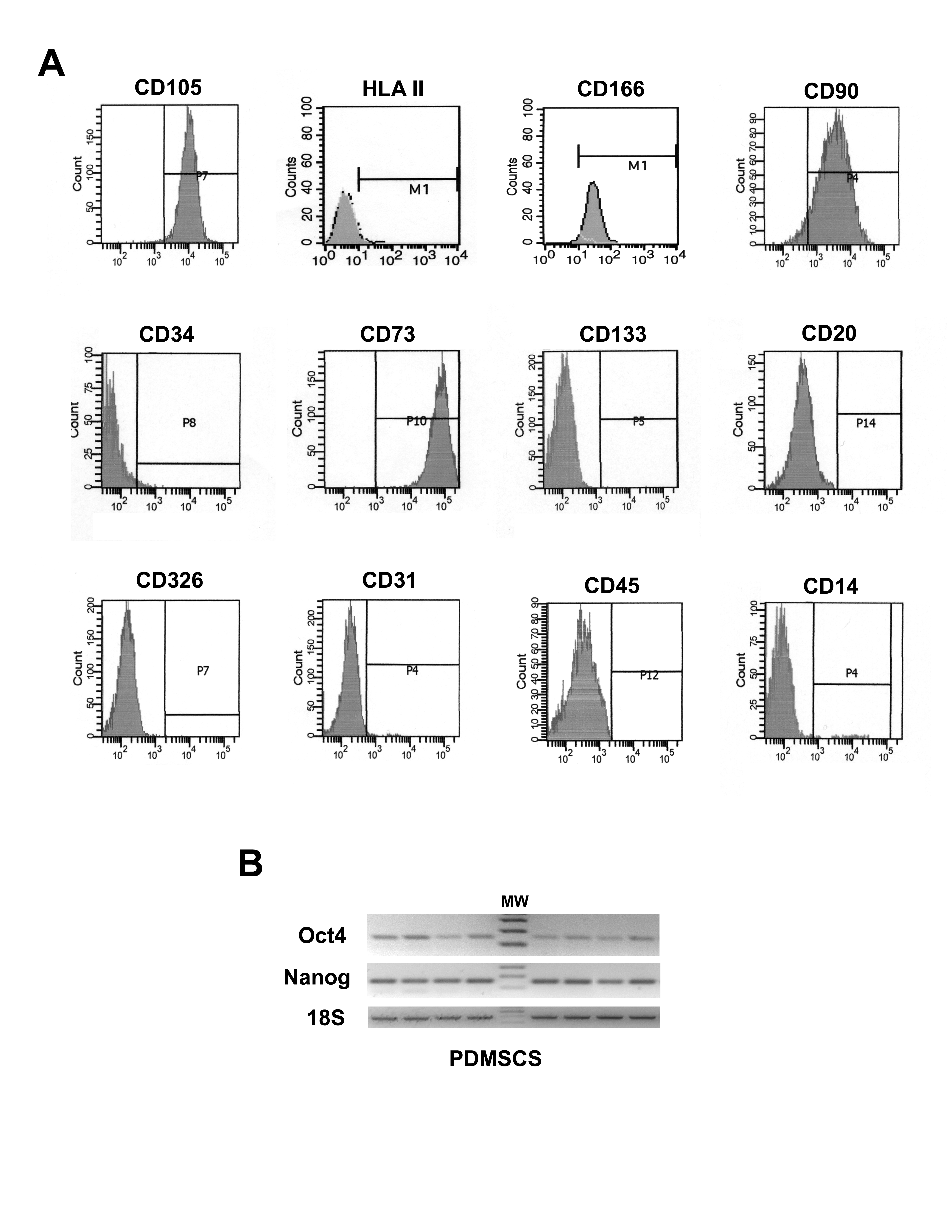

Supplement: Figure S1 — Placenta-derived Mesenchymal Stromal Cells Characterization. A) Representative phenotype of human chorionic PDMSCs at passage 5 as assessed by flow cytometry. All cells were positive for CD166, CD105, CD90, CD73 and negative for HLA II, CD34, CD133, CD20, CD326, CD31, CD45 and CD14, thus displaying proper mesenchymal profile and no contamination from epithelial, hematopoietic, immune or endothelial cells. B) Representative Oct4 and Nanog PCR analysis in control and PE PDMSCs. All cell lines expressed both gene markers of stemness. (TIF) [file pone.0059403.s001.tif]

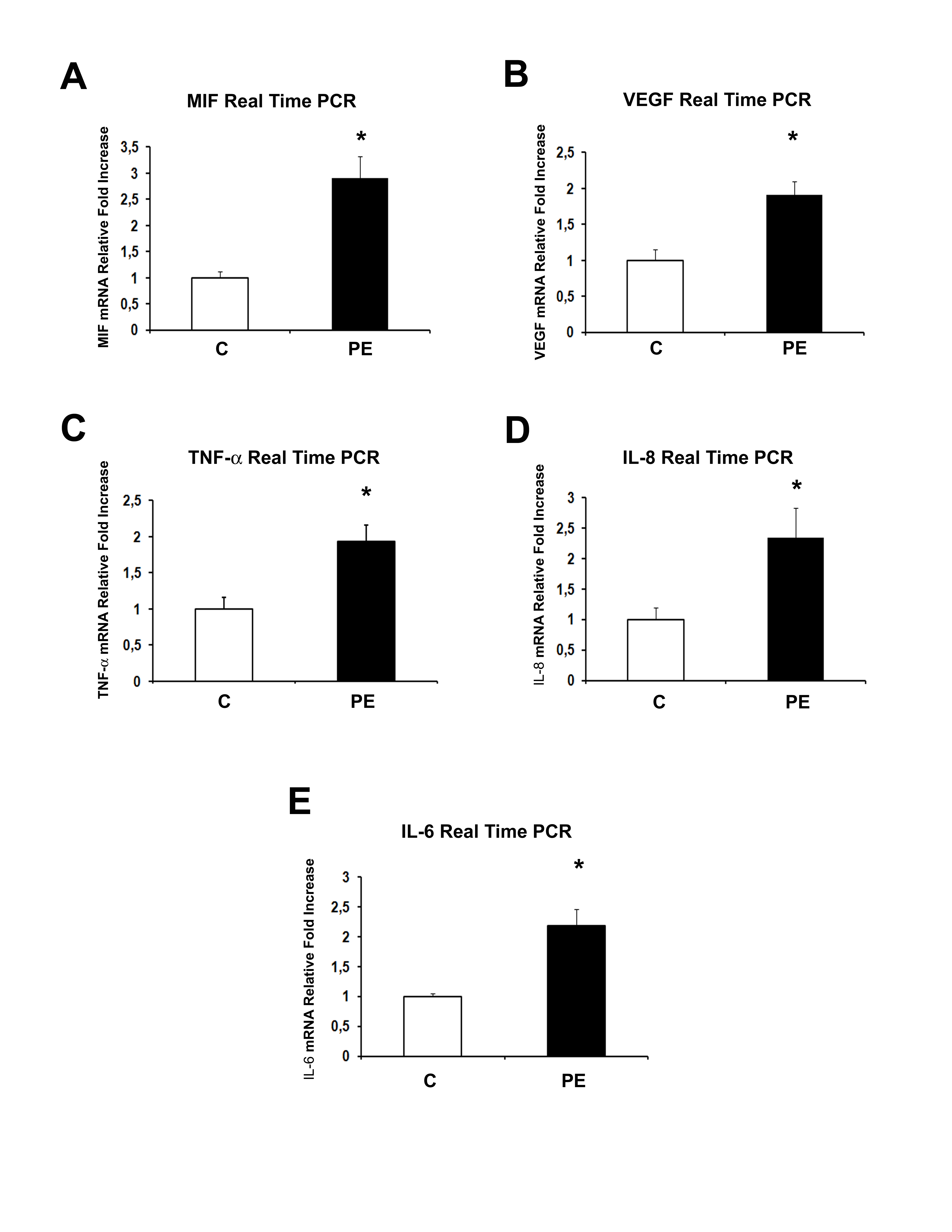

Supplement: Figure S2 — Gene expression levels of key differentially expressed molecules detected by Cytokine Array in normal (C) and preeclamptic (PE) PDMSCs. MIF (A), VEGF (B), TNF-α (C), IL-8 (D) and IL-6 (E) mRNA expression levels in normal and PE-PDMSCs as assessed by Real Time PCR. Statistical significance (*) has been considered as p<0.05. (TIF) [file pone.0059403.s002.tif]

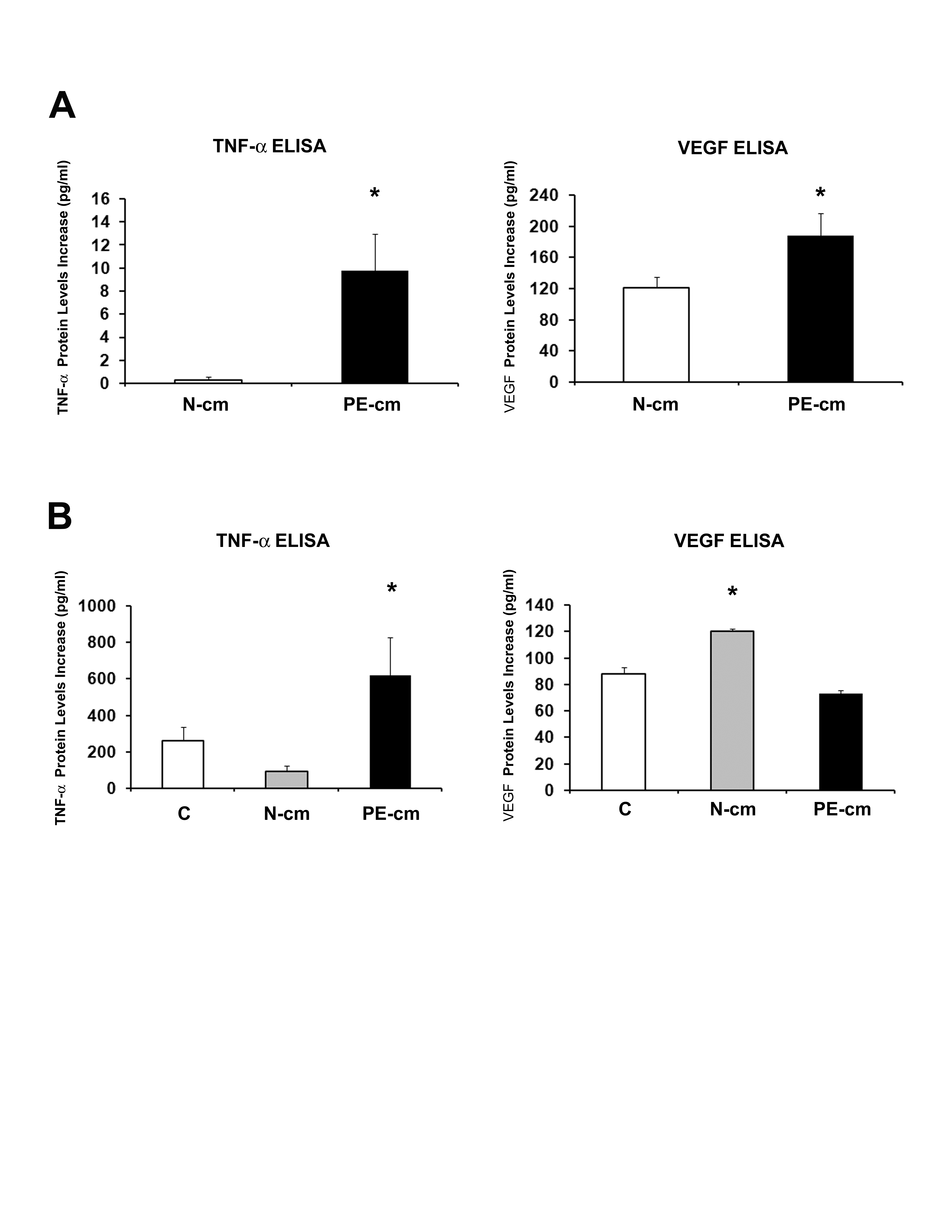

Supplement: Figure S3 — TNF-α and VEGF protein expression in Normal and PE-PDMSCs Conditioned Media and in physiological placental villous explants treated by normal or PE PDMSCs CM as detected by ELISA Assay. (A) TNF-α (left panel) and VEGF (right panel) protein levels in media conditioned by Normal [N-cm] or preeclamptic [PE-cm] PDMSCs. (B) TNF-α (left panel) and VEGF (right panel) protein levels in untreated control explants [C] and explants treated by normal [N-cm] and preeclamptic [PE-cm] PDMSCs conditioned medium. Results are expressed as means ± SE. Statistical significance (*) has been considered as p<0.05. (TIF) [file pone.0059403.s003.tif]
