# Supplementary material for: Pro-Inflammatory Profile of Preeclamptic Placental Mesenchymal Stromal Cells: New Insights into the Etiopathogenesis of Preeclampsia
Source: PLoS One. 2013 Mar 19;8(3):e59403. doi: 10.1371/journal.pone.0059403 (PMC3602067; doi:10.1371/journal.pone.0059403)
Supplement: Table S1 — Cytokines Expression in Normal vs Preeclamptic PDMSCs Conditioned Media. (DOC) [file pone.0059403.s004.doc]

**Table S1. Cytokines Expression in Normal vs Preeclamptic PDMSCs Conditioned Media**

| **Cytokines** | **Normal PDMSC CM (%±SE) n=4** | **PE PDMSC CM (%±SE) n=4** | **p<0.05** | **Cytokines** | **Normal PDMSC CM (%±SE) n=4** | **PE PDMSC CM (%±SE) n=4** | **p<0.05** | **Cytokines** | **Normal PDMSC CM (%±SE) n=4** | **PE PDMSC CM (%±SE) n=4** | **p<0.05** | |
| --- | --- | --- | --- | --- | --- | --- | --- | --- | --- | --- | --- | --- |
| **IL-8** | 30,1±11 | 138,9±9 | ***** | **I309** | 2,0±0,1 | 7,8±0,3 | ***** | **GROa** | 0 | 16,9±7 | |  |
| **Osteopontin** | 27,3±1,5 | 47,8±9 |  | **FGF 9** | 1,9±0,6 | 19,5±1,3 | ***** | **IL4** | 0 | 7,3±2,3 | |  |
| **TIMP-2** | 23,1±3 | 73,4±1 | ***** | **TARC** | 1,7±0,1 | 23,3±4 | ***** | **IL-5** | 0 | 7,1±1,4 | | ***** |
| **NAP-2** | 18,9±0,3 | 30,2±0,1 | ***** | **HGF** | 1,7±0,4 | 6,1±0,5 | ***** | **IL-7** | 0 | 13,4±0,1 | | ***** |
| **MCP-1** | 17,4±2 | 79,8±2 | ***** | **PDGF-bb** | 1,6±0,3 | 25,1±1,7 | ***** | **IL12 p40p70** | 0 | 3,6±0,6 | | ***** |
| **Ostoprotegerin** | 16,8±4 | 39,7±2,4 | ***** | **LEPTIN** | 1,3±0,1 | 24,63,4 | ***** | **IL-13** | 0 | 2,1±0,5 | |  |
| **TGF-b2** | 16,1±0,2 | 53,2±2 | ***** | **MCSF** | 1,1±0,01 | 16,5±2,1 |  | **MCP-2** | 0 | 19,2±3 | | ***** |
| **TIMP-1** | 15,0±1,7 | 37,7±7,2 |  | **TNF-a** | 1,0±0,1 | 21,6±3,3 | ***** | **MCP-3** | 0 | 22,0±3,6 | | ***** |
| **GRO** | 12,9±7,8 | 50,0±8 |  | **FGF 4** | 0 | 11,6±2 |  | **MDC** | 0 | 12,6±1,3 | |  |
| **IP-10** | 12,0±0,1 | 20,4±1,1 | ***** | **IGFBP-4** | 0 | 20,6±1 | ***** | **MIG** | 0 | 12,0±2 | |  |
| **VEGF** | 11,9±3,7 | 33,5±2,5 | ***** | **IL-15** | 0 | 10,9±3 |  | **MIP-1o** | 0 | 2,5±0,1 | | ***** |
| **Oncostatin m** | 10,6±0,1 | 12,2±0.5 |  | **IFN-g** | 0 | 13,7±3,2 |  | **SDF-1** | 0 | 14,9±4,2 | |  |
| **RANTES** | 10,2±1,6 | 20,5±2,2 |  | **IL-16** | 0 | 11,8±2 |  | **TGF-b1** | 0 | 21,1±8 | |  |
| **LIF** | 9,6±0,1 | 18,6±1,4 | ***** | **PARC** | 0 | 15,9±4 | ***** | **EGF** | 0 | 22,7±4,2 | |  |
| **NT-3** | 8,0±3 | 26,9±1,3 | ***** | **MIF** | 0 | 13,6±3,4 | ***** | **IGF-I** | 0 | 6,82,3 | |  |
| **IGFBP-3** | 7,5±2 | 6,6±1,5 |  | **FGF 6** | 0 | 11,2±0,1 | ***** | **Angiogenin** | 0 | 29,8±5 | | ***** |
| **IL-6** | 7,0±0,6 | 107,1±4.4 | ***** | **MIP-3a** | 0 | 3,2±2,2 |  | **Thrombopoietin** | 0 | 7,1±1 | |  |
| **IL1b** | 5,8±0,2 | 13,1±2,4 |  | **TNFb** | 0 | 13,6±5 |  | **BLC** | 0 | 19,6±6 | |  |
| **IGFBP-1** | 4,5±2,2 | 8,7±,5 |  | **Eotaxin** | 0 | 10,9±4 |  | **Fractalkine** | 0 | 20,4±8,5 | |  |
| **IL-2** | 3,9±0,6 | 10,4±3,3 |  | **LIGHT** | 0 | 14,8±7,3 |  | **GCP-2** | 0 | 16,6±4 | |  |
| **GDNF** | 3,9±0,1 | 17,7±4,4 |  | **CKb 8-1** | 0 | 12,5±4,6 |  | **MCP-4** | 0 | 26,1±1,4 | | ***** |
| **BDNF** | 3,5±0,2 | 38,0±5,2 | * | **SCF** | 0 | 12,8±3,6 |  | **NT-4** | 0 | 3,9±1,2 | |  |
| **IL-3** | 3,4±0,3 | 16,2±4 |  | **TGF-b3** | 0 | 14,0±5,6 |  |  |  |  | |  |
| **MIP-1b** | 3,4±0,2 | 25,4±3,5 | ***** | **FGF 7** | 0 | 8,7±0,5 | ***** |  |  |  | |  |
| **IGFBP-2** | 3,2±0,5 | 19,0±0,5 | ***** | **FLT-3 ligand** | 0 | 18,4±4,5 |  |  |  |  | |  |
| **PIGF** | 3,0±0,1 | 20,4±4 | ***** | **Eotaxin 3** | 0 | 7,2±4,1 |  |  |  |  | |  |
| **IL-1a** | 2,8±0,6 | 8,6±0,1 |  | **ENA78** | 0 | 43,3±13,4 | ***** |  |  |  | |  |
| **IL-10** | 2,7±0,6 | 7,1±0,1 |  | **GCSF** | 0 | 3,1±1,4 |  |  |  |  | |  |
| **Eotaxin 2** | 2,0±0,8 | 8,4±1 | ***** | **GMCSF** | 0 | 2,4±1,4 |  |  |  |  | |  |

**PDMSCs:** Placenta Derived Mesenchymal Stromal Cells; **CM:** Conditioned Media; **PE:**Preeclamptic; **SE:** Standard Error; *****: statistically significant (p<0.05)
